# Unraveling the toxicological impact of Bisphenol A exposure on dermatomyositis: An integration of network toxicology and machine learning approaches

**DOI:** 10.1371/journal.pone.0344169

**Published:** 2026-03-30

**Authors:** Lili Cheng, Zhongfu Tang, Ming Li, Chuanbing Huang

**Affiliations:** 1 The First Affiliated Hospital of Anhui University of Chinese Medicine, Hefei, Anhui, China; 2 Institute of Xin'an Medicine and Modernization of Traditional Chinese Medicine, Hefei, Anhui, China; Hamadan University of Medical Sciences, IRAN, ISLAMIC REPUBLIC OF

## Abstract

**Background:**

Dermatomyositis is a common immune-mediated skin disorder whose pathogenesis has not been fully elucidated. Environmental factors play a key role in its onset and progression. Bisphenol A (BPA) is a widespread environmental pollutant known to pose risks to human health. Previous studies have indicated that BPA exposure can disrupt immune function and trigger skin inflammation and autoimmune diseases. However, the role and molecular mechanisms of BPA in dermatomyositis remain unclear. This study aims to systematically elucidate whether and how bisphenol A (BPA) may contribute to the development of dermatomyositis by identifying key toxicological targets and underlying molecular mechanisms through an integrated computational framework.

**Methods:**

The toxicity and pharmacokinetic properties of BPA were predicted using the ProTox 3.0 and ADMElab 2.0 platforms. Network toxicology approaches were employed to explore the pathogenic pathways and mechanisms of BPA in dermatomyositis. Seven machine learning algorithms were applied for cross-validation and identification of core genes. Molecular docking and molecular dynamics (MD) simulations were conducted to evaluate the binding efficiency and stability between BPA and the identified targets.

**Results:**

Integrated results from both prediction platforms revealed that BPA exhibits significant neurotoxicity, nephrotoxicity, hepatotoxicity, skin sensitization, and immunotoxicity. Network toxicology analysis suggested that BPA may influence the progression of dermatomyositis by regulating key factors such as AKT1, BCL2, MMP9, ESR1, and INS, thereby affecting apoptosis, immune-inflammatory responses, pathways in cancer, and the PI3K-Akt signaling pathway. Using LASSO regression, SVM, random forest (RF), GBM, GLM, KNN, and NNET machine learning algorithms, four core genes were identified: SAA1, NACAD, SLC14A1, and MYBPH, all of which were highly expressed in dermatomyositis lesion tissues. Molecular docking studies demonstrated strong binding affinities between BPA and these targets, with the highest binding energy observed for SAA1 at –8.4 kcal/mol. Molecular dynamics simulations further confirmed the high binding stability of the BPA–SAA1 protein–ligand complex. Collectively, these findings suggest that BPA may increase the risk of dermatomyositis by modulating SAA1 protein.

**Conclusion:**

This study identifies SAA1 as a potential target in BPA-induced dermatomyositis, highlighting the impact of BPA on immune regulation and providing a foundation for understanding associated health risks and developing mitigation strategies. Given the limited research on dermatomyositis, further experimental validation is essential to elucidate the pathogenic mechanisms of BPA.

## 1 Introduction

Dermatomyositis (DM) is a chronic idiopathic immune-mediated autoimmune disorder characterized by symmetric proximal myopathy, extensor muscle inflammation, vascular involvement, and distinctive skin rashes. Its incidence ranges from 1.19 to 10 cases per million population [1]. Etiological factors include genetic predisposition, environmental exposures, and immune dysregulation, with environmental factors playing a crucial role in the pathogenesis of the disease [2]. Questionnaire data from large juvenile and adult DM cohorts have indicated numerous environmental exposures associated with DM onset, including ultraviolet radiation, medications (e.g., NSAIDs, antihypertensives, antidepressants), certain infections (such as urinary tract infections and gastroenteritis), and possibly HPV vaccination, ultimately contributing to disease exacerbation [3]. However, the impact of specific environmental contaminants, particularly bisphenol A (BPA), on the pathogenesis of DM remains incompletely understood.

Bisphenol A (BPA), also known as 4,4′-dihydroxy-2,2-diphenylpropane, is an emerging environmental pollutant widely used as a monomer and plasticizer in the production of polycarbonate plastics and epoxy resins, making it one of the most highly demanded chemicals in commercial applications. It is commonly found in bottles, containers, storage items, and food utensils [4]. BPA is ubiquitous in the environment and is associated with widespread human exposure through oral intake, inhalation, dermal absorption, and ocular contact. Accumulating evidence has linked BPA exposure to the development and progression of various diseases, including cervical cancer [5], cardiovascular disorders (Ajibade et al., 2024), and autoimmune conditions [6]. BPA poses health threats by triggering multiple cell death pathways, including necroptosis, pyroptosis, apoptosis, ferroptosis, and autophagy across different cell types [7]. Studies have identified associations between BPA exposure and potential targets in rheumatoid arthritis (RA), systemic lupus erythematosus (SLE), multiple sclerosis (MS), Hashimoto's thyroiditis (HT), inflammatory bowel disease (IBD), type 1 diabetes (T1D), psoriasis, among others [8]. While BPA exposure is known to exacerbate immune responses, the precise mechanisms through which it contributes to DM development remain undetermined.

**Fig 1:**
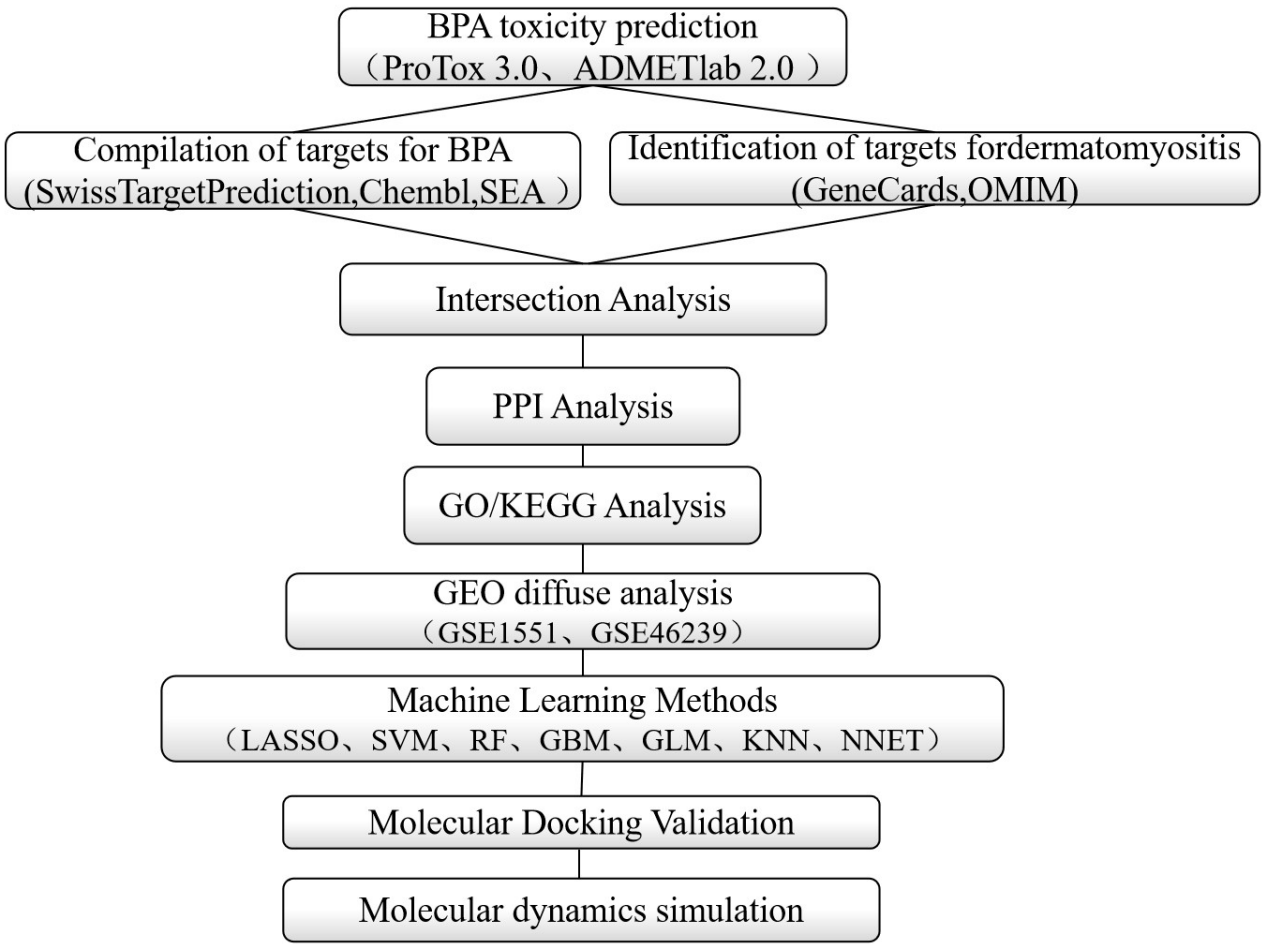
Research flowchart.

In this study, we systematically investigated the potential toxicity and molecular mechanisms of BPA in dermatomyositis using an integrated approach combining network toxicology, machine learning, molecular docking, and molecular dynamics simulations. Network toxicology, which integrates principles from network pharmacology and systems biology, enables the construction of interactive networks connecting compounds, toxicological endpoints, and biological targets. Coupled with big data analytics and multi-omics technologies, this approach helps elucidate complex biological mechanisms [9]. Machine learning, as a major branch of artificial intelligence, offers powerful capabilities for data analysis and predictive modeling. In biological and medical research, it facilitates the processing of large-scale omics data and uncovers underlying patterns and correlations. It has been employed to predict core toxicological targets and construct disease prediction models in myositis research [10]. Furthermore, molecular docking and dynamics simulations were applied to forecast interaction modes and stability between potential ligands and targets [11]. By integrating BPA-related toxicological targets with DM-associated pathogenic genes, this study explores the role of BPA in DM progression (a schematic overview is provided in Fig 1). Our findings provide a theoretical foundation for understanding the toxicological profile and molecular mechanisms of BPA, offering scientific support for environmental risk assessment and the prevention and treatment of related diseases.

## 2 Materials and methods

### 2.1 Toxicity analysis and prediction

To accurately identify potential targets of Bisphenol A (BPA) predicted by multiple databases, the chemical structure and canonical SMILES notation (CC(C)(C1 = CC = C(C = C1)O)C2 = CC = C(C = C2)O) of BPA were first retrieved from the PubChem database (https://pubchem.ncbi.nlm.nih.gov/). The structural information was then input into ProTox 3.0 (https://tox.charite.de) and ADMETlab 3.0 (https://admetlab3.scbdd.com/) for preliminary toxicity prediction based on integrated web-based algorithms. ProTox 3.0 is a virtual platform for predicting the toxicity of small molecules, while ADMETlab 2.0 enables the prediction of absorption, distribution, metabolism, excretion, and toxicity (ADMET) properties of compounds in vivo [12]. These tools provide comprehensive predictions of various toxicological endpoints and pharmacokinetic properties, aiding in the assessment of safety and potential risks associated with BPA.

### 2.2 Collection of BPA targets from multiple databases

Potential targets of BPA in Homo sapiens were retrieved from the ChEMBL (https://www.ebi.ac.uk/chembl/), SEA (https://sea.bkslab.org/), and SwissTargetPrediction (http://swisstargetprediction.ch/) databases. Targets obtained from these three sources were merged, duplicates were removed, and protein names were standardized using the UniProt database [13], resulting in a final set of BPA target genes.

### 2.3 Collection of dermatomyositis-related targets

Dermatomyositis (DM)-related target genes were collected from two authoritative bioinformatics databases: GeneCards (https://www.genecards.org/) and OMIM (https://omim.org/) [14]. After merging and deduplication, a final set of DM-associated genes was obtained.

### 2.4 Construction of protein–protein interaction (PPI) network

Common potential targets of BPA and DM were identified by taking the intersection of their respective target sets. These common targets were then imported into the STRING database (https://cn.string-db.org/) for PPI analysis, with the species limited to Homo sapiens. Results from STRING [15] were imported into Cytoscape (v3.10.2) for visualization. The top ten hub targets were identified using the CytoHubba_Degree algorithm.

### 2.5 GO and KEGG enrichment analysis of target proteins

To explore the biological functions and pathways of potential targets involved in BPA-induced DM, Gene Ontology (GO) and Kyoto Encyclopedia of Genes and Genomes (KEGG) pathway enrichment analyses were performed using the DAVID database (https://davidbioinformatics.nih.gov/). DAVID integrates multiple authoritative data sources to investigate protein interactions, gene functions, and pathways. Results from the GO and KEGG analyses were visualized accordingly [16].

### 2.6 Download of GEO datasets and construction of expression matrix

The gene expression datasets GSE1551 and GSE46239 were downloaded from the GEO database. After annotation, normalization, and merging of the datasets, box plots and PCA analysis were performed. Differential expression analysis was conducted using the limma package, with differentially expressed genes (DEGs) defined as those with |log₂FC| > 2 and an adjusted p-value < 0.05. A differential gene expression matrix was subsequently constructed.

### 2.7 Screening of key target genes using machine learning algorithms

Seven machine learning algorithms—LASSO regression, SVM, random forest (RF), GBM, GLM, KNN, and NNET—were employed to screen for core genes. The DALEX, ggplot2, randomForest, kernlab, xgboost, and fs packages in R were used for core gene selection, generating reverse cumulative distribution plots of residuals, residual box plots, and variable importance plots [17]. The intersection of results from all seven algorithms was taken to establish a core gene set, visualized using a Venn diagram.

### 2.8 Molecular docking of BPA with core targets

The three-dimensional structures of core targets identified through machine learning and network toxicology PPI analysis were downloaded from the PDB database (https://www.rcsb.org/). The 3D structure of BPA was obtained from PubChem (https://pubchem.ncbi.nlm.nih.gov/). Molecular docking was performed using AutoDock Vina to explore potential binding sites. Results were visualized using PyMOL, and binding affinity was evaluated based on binding energy values [18].

### 2.9 Molecular dynamics (MD) simulation

MD simulations were conducted to further analyze and evaluate the binding stability of the receptor–ligand complexes. Complexes with binding energies ≤ –5.0 kcal/mol were ranked, and the best-docked complex was selected for MD simulation. The protein and ligand were separated, and the Sobtop software (http://sobereva.com/soft/Sobtop) was used to generate ligand topology files. Simulations were performed using Gromacs 2022. Force field parameters for the protein and ligand were obtained using the amber14sb and GAFF2 force fields, respectively, via the pdb2gmx tool and the AutoFF web server. The system was solvated in a cubic TIP3P water box with a 1 nm margin. Ions were added using gmx genion to neutralize the system. Long-range electrostatic interactions were handled using the Particle Mesh Ewald (PME) method with a cutoff of 1 nm. Bond constraints were applied using the SHAKE algorithm, and the integration step was set to 1 fs using the Verlet leapfrog algorithm. Simulations were run under NPT conditions at 310 K for 100 ns. The gmx rms, gmx rmsf, gmx hbond, gmx gyrate, and gmx sasa tools were used to calculate the root mean square deviation (RMSD), root mean square fluctuation (RMSF), hydrogen bonds (HBonds), radius of gyration (Rg), and solvent accessible surface area (SASA), respectively [19].

## 3 Results

### 3.1 Toxicity prediction of BPA

**Fig 2:**
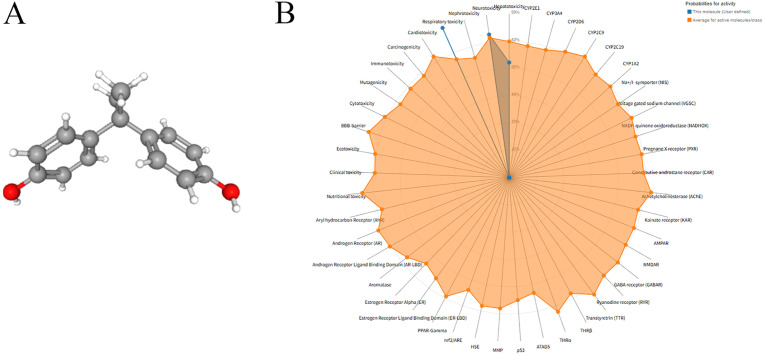
Molecular structure, toxicity profile radar chart of BPA. 3D molecular structure of BPA; (B) Radar chart of BPA toxicity distribution predicted by ProTox 3.0.

The three-dimensional chemical structure of Bisphenol A (BPA) (Fig 2A) and its canonical SMILES notation (CC(C)(C1 = CC = C(C = C1)O)C2 = CC = C(C = C2)O) were retrieved from the PubChem database. Toxicity prediction performed using the ProTox 3.0 platform indicated that BPA exhibits organ toxicity including hepatotoxicity, neurotoxicity, nephrotoxicity, respiratory toxicity, and cardiotoxicity. Endpoint toxicities identified were carcinogenicity, immunotoxicity, mutagenicity, and cytotoxicity (Table 1, Fig 2B). Predictions from the ADMETlab 3.0 platform further revealed that BPA toxicity primarily includes drug-induced neurotoxicity, HEK293 cytotoxicity, A549 cytotoxicity, and skin sensitization, among others (Table 2).

**Table 1:** PROTox – Prediction of toxicity of chemicals.

Classification	Target	Probability
Organ toxicity	Hepatotoxicity	0.85
Organ toxicity	Neurotoxicity	0.64
Organ toxicity	Nephrotoxicity	0.80
Organ toxicity	Respiratory toxicity	0.79
Organ toxicity	Cardiotoxicity	0.87
Toxicity end points	Carcinogenicity	0.90
Toxicity end points	Immunotoxicity	0.99
Toxicity end points	Mutagenicity	0.98
Toxicity end points	Cytotoxicity	0.90

**Table 2:** Toxicophore Rules.

Classification	Probability
hERG Blockers(10um)	0.85
Skin Sensitization	0.64
Eye Corrosion	0.80
Eye Irritation	0.79
A549 Cytotoxicity	0.87
Hek293 Cytotoxicity	0.90
Drug-induced Neurotoxi city	0.99

### 3.2 Identification of BPA targets associated with dermatomyositis

**Fig 3:**
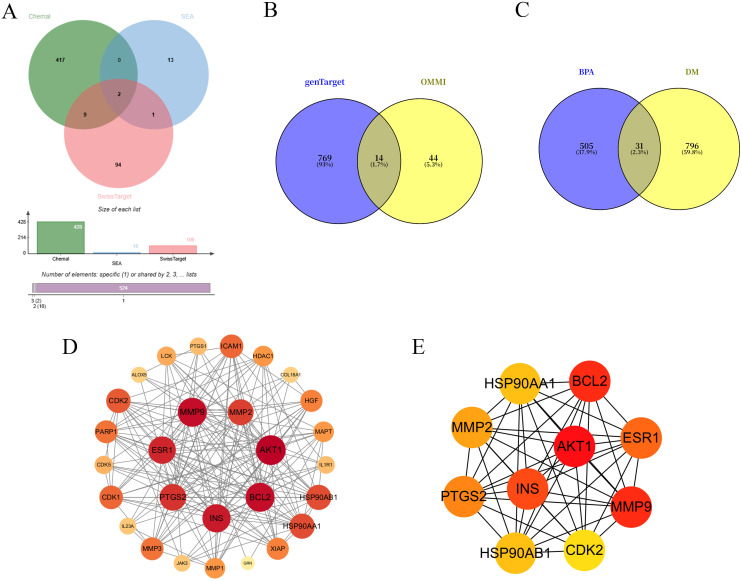
Visualization of target genes for BPA and dermatomyositis (DM). (A) Venn diagram of BPA target genes; (B)Venn diagram of DM target genes; (C) Venn diagram illustrating the intersection of BPA and DM target genes; (D) PPI network of the overlapping targets;(E) Core targets identified using the CytoHubba_Degree algorithm.

A total of 428, 16, and 106 potential target genes of BPA were retrieved from the ChEMBL, SEA, and SwissTargetPrediction databases, respectively. After merging and removing duplicates, 538 unique BPA target genes were obtained (Fig 3A). Meanwhile, 783 and 58 dermatomyositis (DM)-related target genes were collected from the GeneCards and OMIM databases, respectively. Following the removal of redundant entries, a total of 827 DM-associated target genes were identified (Fig 3B).

### 3.3 Protein-protein interaction network and identification of core targets

The potential target genes of BPA and dermatomyositis (DM) were visualized as shown in Fig 3. Intersection analysis identified 31 common target genes (Fig 3C), which are considered potential key targets through which BPA may influence DM. These 31 common targets were imported into the STRING database to construct a protein-protein interaction (PPI) network. The resulting network was visualized using Cytoscape software, revealing complex interactions among the potential targets (Fig 3D). Node color intensity reflects the degree of connectivity, with darker shades indicating higher degree values. Using the CytoHubba_Degree algorithm, the top ten hub targets were identified, including AKT1, BCL2, MMP9, ESR1, and INS, among others (Fig 3E).

### 3.4 GO and KEGG enrichment analysis

**Fig 4:**
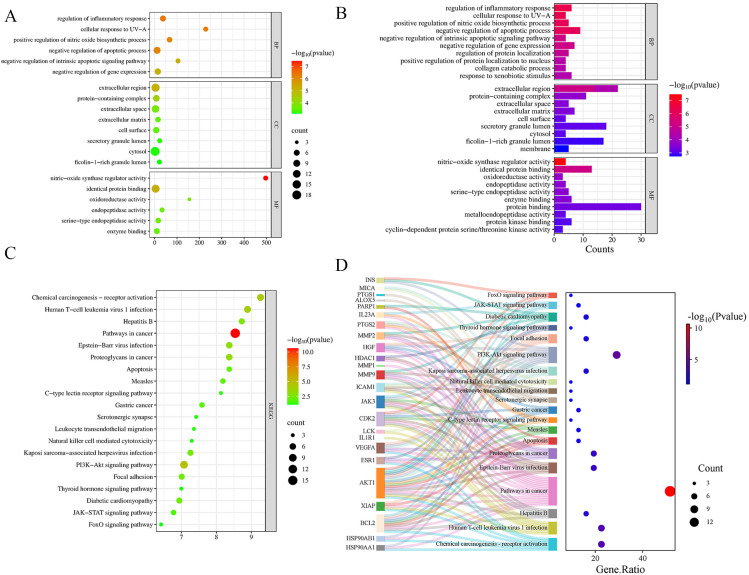
GO and KEGG enrichment analysis of potential targets. (A) Bubble plot of GO enrichment analysis for potential targets; (B) Histogram of GO enrichment analysis showing the top 10 terms in biological process (BP), cellular component (CC), and molecular function (MF); (C) Bubble plot of the top 20 KEGG pathways enriched for potential targets; (D) Sankey diagram of KEGG enrichment analysis for core targets.

The 31 common target genes were imported into the DAVID database for Gene Ontology (GO) and Kyoto Encyclopedia of Genes and Genomes (KEGG) pathway enrichment analyses. The GO analysis revealed that these target genes were significantly associated with several biological processes (BP), including regulation of inflammatory response, cellular response to UV-A, positive regulation of nitric oxide biosynthetic process, and negative regulation of apoptotic process. For cellular component (CC), the targets were mainly enriched in the extracellular region, protein-containing complex, extracellular space, and cell surface. Molecular functions (MF) included nitric-oxide synthase regulator activity, identical protein binding, oxidoreductase activity, and endopeptidase activity (Fig 4A, 4B). KEGG pathway analysis indicated significant enrichment in pathways such as Pathways in cancer, PI3K-Akt signaling pathway, Chemical carcinogenesis – receptor activation, JAK-STAT signaling pathway, and FoxO signaling pathway (Fig 4C, 4D). These results suggest that BPA may influence the progression of dermatomyositis by mediating processes such as apoptosis and immune-inflammatory responses.

### 3.5 Screening results from GEO data analysis

The GSE1551 and GSE46239 datasets were annotated, normalized, and merged.The results of the relevant analyses are shown in S1 Table and S1 Fig. Box plots before and after batch normalization are presented. Principal component analysis (PCA) was performed to evaluate the distribution of samples before and after normalization. Differential gene expression analysis was conducted using the limma package, and the results are visualized through a heatmap and a volcano plot of the differentially expressed genes (DEGs). A final matrix of differentially expressed genes was subsequently obtained, the results of the relevant analyses are shown in S2 Table.

### 3.6 Screening of key genes using machine learning algorithms

**Fig 5:**
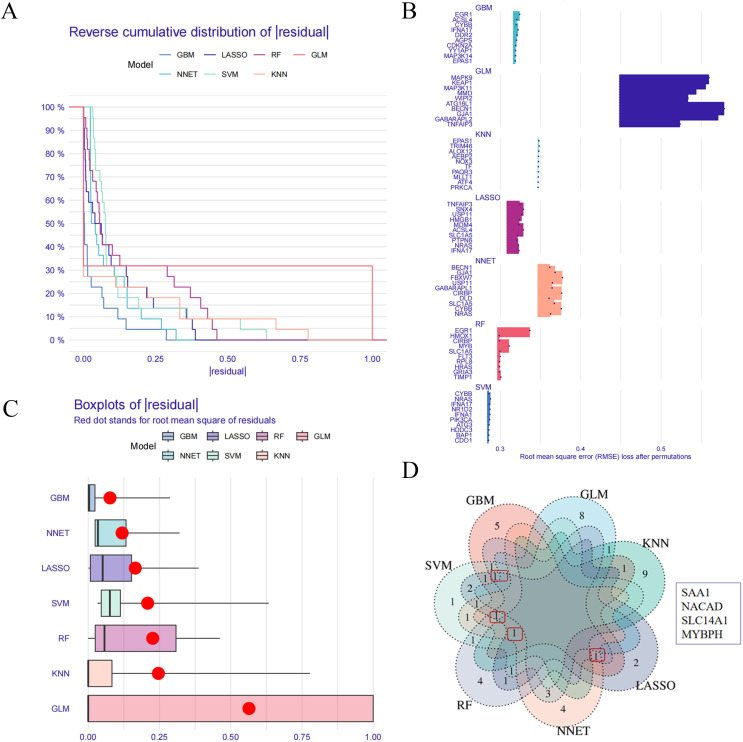
Screening of key genes in DM using machine learning methods. Absolute residual plot across various machine learning algorithms; (B) Box plot of residuals from different machine learning algorithms; (C) Core genes identified by each machine learning algorithm; (D) Venn diagram of core genes derived from multiple machine learning algorithms.

Seven machine learning algorithms—GBM, LASSO, RF, GLM, KNN, NNET, and SVM—were employed to evaluate key genes in the integrated GSE1551 and GSE46239 datasets. The absolute residual distribution and residual box plots are shown in Fig 5A and 5C, respectively. The top ten core genes identified by each algorithm are summarized as follows (Fig 5B).Intersection analysis of the results from all seven algorithms identified four key genes: SAA1, NACAD, SLC14A1, and MYBPH (Fig 5D).

### 3.7 Molecular docking of BPA with key target proteins

**Fig 6:**
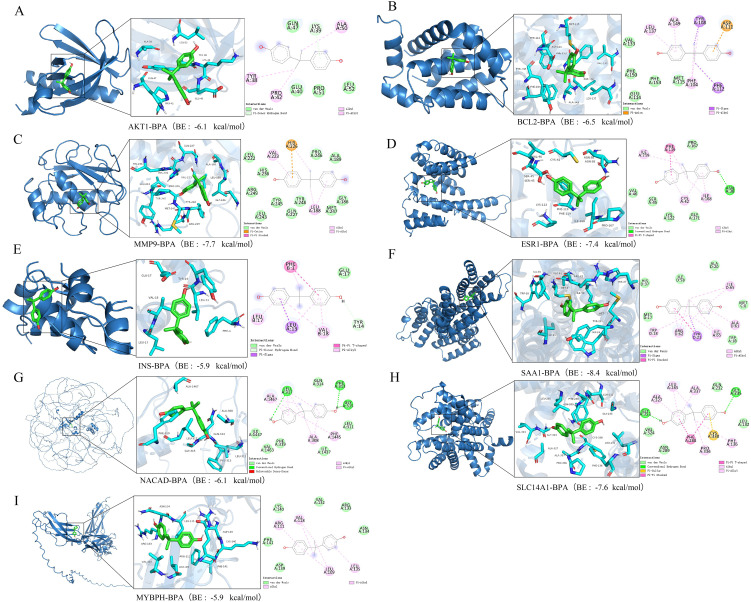
Molecular docking of BPA with key target proteins. (A) AKT1-BPA; (B) BCL2-BPA; (C) MMP-9-BPA; (D) ESR1-BPA; (E) INS-BPA; (F) SAA1-BPA; (G) NACAD-BPA; (H) SLC14A1-BPA; (I) MYBPH-BPA.

To investigate the interactions between BPA and key target proteins—namely AKT1, BCL2, MMP9, ESR1, INS, SAA1, NACAD, SLC14A1, and MYBPH—molecular docking analysis was performed. A binding energy threshold of –5 kcal/mol was applied to confirm stable binding affinity. The binding conformations for each complex were visualized using PyMOL (Fig 6). The results demonstrated strong binding affinities between BPA and all nine targets. When arranged from the strongest to the weakest binding based on binding energy, BPA showed the highest affinity for SAA1 (–8.4 kcal/mol), followed by MMP9 (–7.7 kcal/mol), SLC14A1 (–7.6 kcal/mol), ESR1 (–7.4 kcal/mol), BCL2 (–6.5 kcal/mol), AKT1 and NACAD (both –6.1 kcal/mol), and INS and MYBPH (both –5.9 kcal/mol) (Fig 6A–6I). These findings indicate that BPA exhibits the highest binding stability with SAA1, suggesting SAA1 may be a critical protein through which BPA influences the development of dermatomyositis.

### 3.8 Molecular dynamics simulation of BPA with SAA1

**Fig 7:**
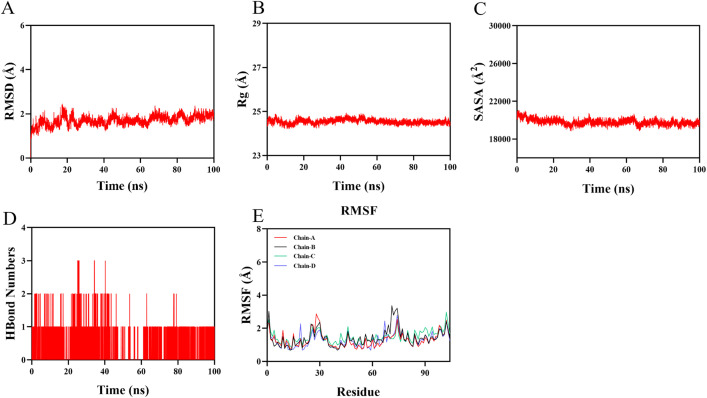
Molecular dynamics simulation of the SAA1–BPA complex. (A) Root mean square deviation (RMSD) of the SAA1–BPA complex over time; **(B)** Radius of gyration (Rg) of the complex over time; **(C)** Solvent accessible surface area (SASA) of the complex over time; **(D)** Number of hydrogen bonds (H-bonds) between SAA1 and BPA over time; **(E)** Root mean square fluctuation (RMSF) of the SAA1–BPA complex.

The root mean square deviation (RMSD) is a key metric for assessing the conformational stability of protein-ligand complexes, reflecting the deviation of atomic positions from their initial state. Lower RMSD values indicate greater conformational stability. The equilibrium of the simulation system was evaluated using RMSD analysis. As shown in Fig 7A, the BPA–SAA1 complex reached equilibrium after 90 ns, with fluctuations stabilizing around 1.9 Å, demonstrating high stability upon binding.The radius of gyration (Rg), which reflects the overall compactness of the protein structure, showed slight fluctuations during the simulation, indicating conformational changes in the protein-ligand complex over time (Fig 7B).The solvent accessible surface area (SASA), used to evaluate changes in protein surface accessibility, exhibited minor variations throughout the simulation (Fig 7C). This suggests that ligand binding influences the micro-environment at the binding site, leading to moderate alterations in SASA.The number of hydrogen bonds formed between BPA and SAA1 during the simulation ranged from 0 to 3, with an average of approximately 1 hydrogen bond maintained throughout most of the trajectory (Fig 7D). This indicates consistent and favorable hydrogen bonding interactions between the ligand and target protein.The root mean square fluctuation (RMSF), which measures the flexibility of individual amino acid residues, showed relatively low values (mostly below 2.3 Å) across the protein structure (Fig 7E), suggesting reduced flexibility and high stability of the complex. The BPA–SAA1 complex exhibited strong binding stability, supported by consistent hydrogen bonding and low structural fluctuations, confirming a favorable interaction between the ligand and target protein. SAA1 not only exhibited the strongest binding affinity with BPA in molecular docking but also demonstrated the highest structural stability during molecular dynamics simulations. The consistency between docking scores and dynamic stability highlights SAA1 as a robust interaction partner of BPA rather than a transient or model-dependent hit.

## 4 Discussion

To investigate the potential causal relationship between environmental pollution and dermatomyositis (DM), this study focused on bisphenol A (BPA), a widely used plasticizer. Predictions from two major toxicity assessment platforms indicated that BPA exhibits significant hepatotoxicity, nephrotoxicity, skin sensitization, and immunotoxicity. Animal studies have also demonstrated the immunopathological potential of BPA [20,21]. Our investigation into the effects of BPA on DM is grounded in a solid theoretical foundation.

Bisphenol substitutes such as bisphenol S (BPS) and bisphenol F (BPF) have increasingly replaced BPA in “BPA-free” products, yet growing evidence suggests that these analogues can also exert immunomodulatory effects. Reviews of BPA analogues summarize impacts on both innate and adaptive immunity and describe pro-inflammatory signaling changes consistent with immune-mediated diseases. Experimental data indicate that exposure to BPA, BPS, or BPF can modulate NF-κB–related cytokine programs (e.g., IL-1β, IL-6, TNFα, IFNγ) and that pathway inhibition attenuates these effects, supporting shared inflammatory signaling nodes across bisphenols [22,23]. In human studies of autoimmunity (e.g., SLE), elevated exposure to bisphenol analogues (including BPF, and related bisphenols) has been associated with disease risk, suggesting that bisphenol substitutes should be considered in environmental risk assessments [6]. However, dermatomyositis-specific studies for BPS/BPF remain limited, and extrapolation to DM should be made cautiously.

Our study provides substantial evidence that BPA may act as a risk factor in the development of DM. Based on network toxicology, BPA is predicted to modulate multiple key proteins and regulatory factors in DM primarily through pathways such as Pathways in cancer, PI3K-Akt signaling, Chemical carcinogenesis – receptor activation, JAK-STAT signaling, and FoxO signaling. These findings suggest that BPA may influence the progression of DM by mediating processes including apoptosis and immune-inflammatory responses. Although this study provides mechanistic evidence supporting a potential association between BPA and dermatomyositis, it is important to emphasize that BPA should be regarded as an environmental risk factor rather than a direct causative agent. BPA exposure in the general population is widespread and typically occurs at low but chronic levels through diet, inhalation, and dermal contact. Accumulating evidence indicates that such exposure can disrupt immune homeostasis, enhance pro-inflammatory signaling, and increase susceptibility to autoimmune diseases, particularly in genetically or immunologically predisposed individuals. However, at present, no disease-specific exposure threshold for dermatomyositis has been established, and the risk is likely to be modulated by cumulative exposure, individual susceptibility, and co-existing environmental factors.

To date, no *in vivo* study has specifically examined the role of BPA exposure in dermatomyositis models. This reflects a broader limitation in the field, as disease-specific environmental exposure models for dermatomyositis are still lacking. Multiple animal studies have demonstrated that BPA exposure can induce immune imbalance, promote pro-inflammatory cytokine production, and activate interferon-related signaling pathways, all of which are central features of dermatomyositis pathogenesis. Although direct in vivo validation in dermatomyositis is currently unavailable, existing animal evidence supports the biological plausibility of our findings.

Research into the pathogenesis of DM has shown that skeletal muscle inflammation promotes muscle atrophy by inhibiting PI3K-Akt-mediated myogenic signaling, which involves AMPK, p38 MAPK, JNK, mTOR, and IGF-1 activation [24]. The JAK/STAT pathway stimulates the expression of interferon-induced genes and serves as a master regulator of inflammation and cell death. Its central role in interferon signaling is particularly relevant to DM pathogenesis [25]. A systematic review of JAK inhibitors in DM, encompassing 28 publications and 61 patients with refractory skin lesions, reported dermatological improvement in all cases following JAK inhibitor therapy [26]. FoxO transcription factors are conserved regulators of diverse cellular processes, including cell cycle control, redox balance, protein homeostasis, apoptosis, metabolism, and DNA damage repair. These factors undergo extensive post-translational modifications such as phosphorylation, acetylation, ubiquitination, and methylation [27]. The interconnected PI3K-Akt, JAK-STAT, and FoxO signaling networks play decisive roles in maintaining skeletal muscle homeostasis and regulating the differentiation, proliferation, and regeneration of muscle cells [28,29]. These mechanisms support a relatively high risk of BPA contributing to DM onset.

Through degree-based ranking, key candidate targets of BPA were identified, including AKT1, BCL2, MMP9, ESR1, and INS. DM is an autoimmune inflammatory myopathy characterized primarily by skin erythema and muscle weakness. Its pathogenesis involves aberrant activation of innate and adaptive immunity, microangiopathy, and dysregulation of cytokine and chemokine networks [30]. AKT1 is a central component of the PI3K/AKT/mTOR pathway, influencing cell survival, proliferation, metabolism, and angiogenesis [31]. In autoimmune contexts, hyperactivation of AKT signaling can inhibit lymphocyte apoptosis, leading to abnormal survival and persistent activation of immune cells that attack self-tissues [32]. DM exhibits a strong type I interferon (IFN-I) signature [33], and evidence suggests that IFN-I can activate the PI3K/AKT pathway, which may further amplify inflammatory responses, forming a positive feedback loop that perpetuates the disease [34]. BCL2, an important anti-apoptotic protein, promotes cell survival by inhibiting mitochondrial apoptosis [35]. Similar mechanisms have been implicated in other autoimmune diseases such as systemic lupus erythematosus [36], and may also operate in DM. Elevated levels of MMP9 have been detected in the serum and muscle tissues of DM patients and correlate with disease activity, suggesting its utility as a biomarker [37]. ESR1, the primary estrogen receptor, mediates various biological effects of estrogen, including immunomodulation. Accumulating evidence indicates that sex hormones play a critical role in shaping immune responses, with androgens generally exerting immunosuppressive effects. Reduced androgen-mediated immune regulation in females has been proposed as one of the biological mechanisms contributing to the higher incidence of autoimmune disorders in women [38,39].

Machine learning algorithms offer unique advantages in handling high-dimensional data and complex models [40]. Cross-validation across multiple algorithms enhances the reliability and accuracy of the results. Using seven machine learning methods (LASSO, SVM, RF, GBM, GLM, KNN, NNET), we analyzed GEO data and identified the top ten core targets from each algorithm. Intersection analysis revealed four key genes: SAA1, NACAD, SLC14A1, and MYBPH. All four genes were consistently identified because they exhibit pronounced differential expression in dermatomyositis tissues and contribute substantially to the discrimination between disease and control samples across diverse algorithmic frameworks. SAA1 is an acute-phase protein primarily produced by the liver in response to inflammatory cytokines such as IL-1, IL-6, and TNF-α [41]. Multiple genome-wide association studies (GWAS) have consistently linked specific variants in the SAA1 gene (particularly rs1059559 and rs12218) to increased DM susceptibility in East Asian populations [42]. SAA1 itself acts as a potent pro-inflammatory factor that can activate the NLRP3 inflammasome, leading to IL-1β release and establishing a positive feedback loop that sustains inflammation [43]. Upregulation of SAA1 has been observed in the skin and muscle lesions of DM patients, indicating its direct involvement in local tissue inflammation and damage [44]. NACAD and SLC14A1 have also been identified as genetic risk loci for DM in GWAS [42]. MYBPH encodes myosin-binding protein H, which is expressed in slow-twitch skeletal muscle fibers and contributes to the maintenance of muscle structure and regulation of contraction [45,46].

Molecular docking was performed to simulate the binding affinity between BPA and the key target proteins. The results indicated particularly favorable binding with SAA1, which was further corroborated by molecular dynamics (MD) simulations showing high stability. These findings suggest a strong interaction between BPA and SAA1. Increased intake of BPA may enhance its binding to SAA1, affect the expression of SAA1 protein, trigger inflammatory signaling pathways, and ultimately exacerbate the development of DM. Previous research has shown that SAA1 binds to Toll-like receptors (TLR) 2 and 4 in muscle, activating the canonical NF-κB p65 pathway and leading to phosphorylation and nuclear translocation of NF-κB p65/RelA. In the nucleus, NF-κB binds to NF-κB response elements (NRE), promoting the expression and secretion of pro-inflammatory cytokines such as IL-6. IL-6, in turn, induces further expression and secretion of SAA1, forming a positive feedback loop that contributes to muscle atrophy [47]. SAA1 has been proposed as a prognostic marker and therapeutic target in rheumatoid arthritis [48,49]. In summary, BPA may increase the risk of DM by modulating SAA1 protein. Research on the relationship between SAA1 and DM remains limited, necessitating further validation using diverse technical and methodological approaches.

From a public health perspective, the widespread environmental contamination and chronic low-dose exposure to BPA raise important concerns. BPA exposure occurs through multiple routes, including diet, inhalation, and dermal contact, resulting in sustained internal exposure across the lifespan. Although typical exposure levels are often below acute toxicity thresholds, increasing evidence indicates that long-term, low-dose exposure can disrupt immune homeostasis and promote chronic inflammatory states. Such effects may increase susceptibility to immune-mediated diseases, particularly in genetically predisposed or immunologically vulnerable populations. Our findings suggest that BPA should be regarded as an environmental risk modifier rather than a direct etiological agent, highlighting the need for strengthened regulatory policies, continuous biomonitoring, and preventive strategies aimed at reducing cumulative exposure.

The novelty of this study lies in the incorporation of MD simulations and machine learning techniques. Certain limitations should be acknowledged. Further experimental validation is required to substantiate the proposed role of BPA in dermatomyositis. At the cellular level, human skeletal muscle cells, immune cells, and skin-relevant cell types should be used to examine whether BPA exposure alters SAA1 expression and activates downstream inflammatory pathways such as TLR/NF-κB, IL-6, and JAK–STAT signaling. At the organismal level, controlled BPA exposure in interferon-driven or inflammatory myopathy–like animal models could be used to assess whether environmental exposure modulates disease susceptibility or severity. At the clinical level, observational cohort studies integrating BPA exposure biomarkers with SAA1 expression and disease activity indices in dermatomyositis patients would be essential to establish translational relevance. Currently, research on DM is relatively scarce, underscoring the need for increased public awareness and scientific attention. This study establishes a foundational basis for exploring the potential role of BPA in DM and provides theoretical support for future epidemiological investigations. It also offers robust scientific evidence for the development of food safety policies and regulatory guidelines concerning plasticizers.

## 5 Conclusion

In summary, this integrated network toxicology and machine learning study demonstrates that bisphenol A (BPA) may contribute to the development of dermatomyositis by modulating key proteins—including AKT1, BCL2, MMP9, ESR1, INS, SAA1, NACAD, SLC14A1, and MYBPH—leading to dysregulation of the PI3K-AKT, JAK-STAT, and FoxO signaling pathways, which in turn promotes apoptosis and immune-inflammatory responses. The binding stability between BPA and SAA1 was further validated through molecular docking and molecular dynamics simulations. Our findings underscore the potential immunotoxicity of BPA as a plasticizer in the context of dermatomyositis, laying a foundation for future investigations into its immunotoxic potential and providing new insights for the development of safety policies and regulatory guidelines concerning BPA exposure.

## Supporting information

S1 Fig Analysis of GEO datasets. (TIF)

S1 Table Gene expression matrix. (XLS)

S2 Table Differentially expressed genes (DEGs) analysis results. (XLS)

S1 File Supplementary material machine learning algorithm code. (DOC)
