# Supplementary figures and images for: Unraveling the toxicological impact of Bisphenol A exposure on dermatomyositis: An integration of network toxicology and machine learning approaches
Source: PLoS One. 2026 Mar 30;21(3):e0344169. doi: 10.1371/journal.pone.0344169 (PMC13035142; doi:10.1371/journal.pone.0344169)

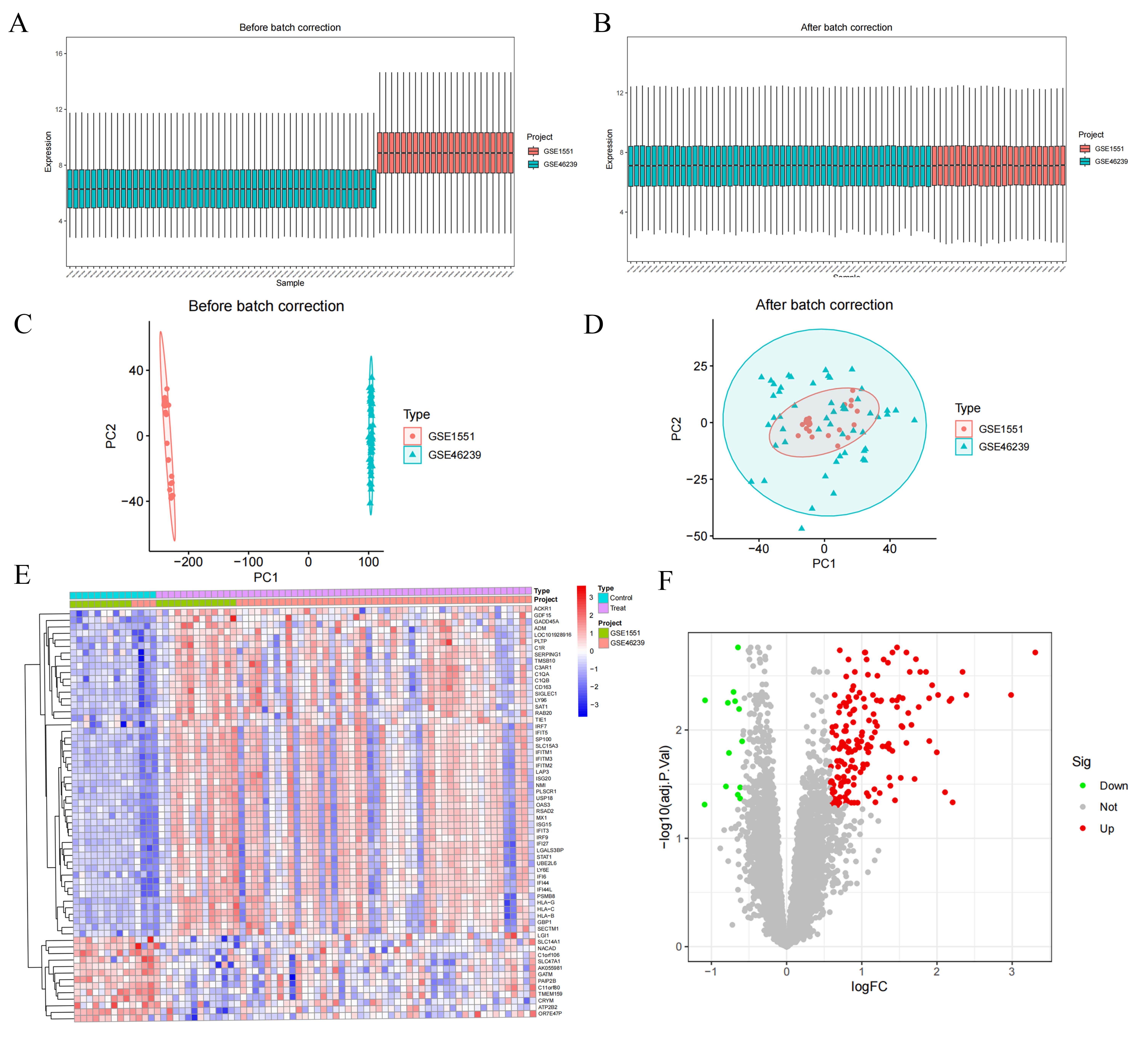

Supplement: S1 Fig — (TIF) [file pone.0344169.s001.tif]
