# Supplementary material for: Unraveling the toxicological impact of Bisphenol A exposure on dermatomyositis: An integration of network toxicology and machine learning approaches
Source: PLoS One. 2026 Mar 30;21(3):e0344169. doi: 10.1371/journal.pone.0344169 (PMC13035142; doi:10.1371/journal.pone.0344169)
Supplement: S1 File — (DOC) [file pone.0344169.s004.doc]

#install.packages("caret")

#install.packages("DALEX")

#install.packages("ggplot2")

#install.packages("randomForest")

#install.packages("kernlab")

#install.packages("pROC")

#install.packages("xgboost")

#install.packages("fs")

#引用包

library(caret)

library(DALEX)

library(ggplot2)

library(randomForest)

library(kernlab)

library(xgboost)

library(pROC)

library(fs)

#创建一个文件夹

dir_create("结果")

#设置种子，重复分析结果

set.seed(123)

#设置工作目录

setwd("C:\\Users\\ABC\\Desktop\\网络毒理学联合机器学习分析\\7.8种机器学习算法")

# 读取文件

expression_matrix<-read.csv("表达矩阵.csv",row.names = 1)

core_genes <- read.csv("核心基因集.csv")[, 2] # 提取第二列的基因名称

sample_classification<-read.csv("样本信息.csv",row.names = 1)

#提取核心基因的表达量：

data <- expression_matrix[core_genes, , drop = FALSE]

#

# 去除表达为NA的值

data <- na.omit(data)

#转置数据

data <- t(data)

#增加分类信息

data <- as.data.frame(data)

data$Type <- sample_classification[rownames(data), "title"]

#对数据进行分组

#p=0.7 指定了训练集占整个数据集的比例，这里设为 70%。

#也就是说，70% 的数据将被分配到训练集中，剩下的 30% 则分配到测试集中。

inTrain<-createDataPartition(y=data$Type, p=0.7, list=F)

train<-data[inTrain,]

test<-data[-inTrain,]

#RF随机森林树模型

control=trainControl(method="repeatedcv", number=5, savePredictions=TRUE)

mod_rf = train(Type ~ ., data = train, method='rf',

trControl = control)

#SVM机器学习模型

mod_svm=train(Type ~., data = train, method = "svmRadial",

prob.model=TRUE, trControl=control)

#GLM模型

mod_glm=train(Type ~., data = train, method = "glm",

family="binomial", trControl=control)

#GBM模型

mod_gbm=train(Type ~., data = train, method = "gbm",

trControl=control)

#KNN模型

mod_knn=train(Type ~., data = train, method = "knn",

trControl=control)

#NNET模型

mod_nnet=train(Type ~., data = train, method = "nnet",

trControl=control)

#Lsso模型

mod_lasso=train(Type ~., data = train, method = "glmnet",

trControl=control)

#定义预测函数

p_fun=function(object, newdata){

predict(object, newdata=newdata, type="prob")[,2]

}

yTest=ifelse(test$Type=="Control", 0, 1)##注意更改自己的分类类型

#RF随机森林树模型预测结果

explainer_rf=explain(mod_rf, label = "RF",

data = test, y = yTest,

predict_function = p_fun,

verbose = FALSE)

mp_rf=model_performance(explainer_rf)

#SVM机器学习模型预测结果

explainer_svm=explain(mod_svm, label = "SVM",

data = test, y = yTest,

predict_function = p_fun,

verbose = FALSE)

mp_svm=model_performance(explainer_svm)

#GLM模型预测结果

explainer_glm=explain(mod_glm, label = "GLM",

data = test, y = yTest,

predict_function = p_fun,

verbose = FALSE)

mp_glm=model_performance(explainer_glm)

###GBM模型预测结果

explainer_gbm=explain(mod_gbm, label = "GBM",

data = test, y = yTest,

predict_function = p_fun,

verbose = FALSE)

mp_gbm=model_performance(explainer_gbm)

#KKNN模型预测结果

explainer_knn=explain(mod_knn, label = "KNN",

data = test, y = yTest,

predict_function = p_fun,

verbose = FALSE)

mp_knn=model_performance(explainer_knn)

#nnet模型预测结果

explainer_nnet=explain(mod_nnet, label = "NNET",

data = test, y = yTest,

predict_function = p_fun,

verbose = FALSE)

mp_nnet=model_performance(explainer_nnet)

#lasso模型预测结果

explainer_lasso=explain(mod_lasso, label = "LASSO",

data = test, y = yTest,

predict_function = p_fun,

verbose = FALSE)

mp_lasso=model_performance(explainer_lasso)

#绘制四种方法的残差反向累计分布图

#下面的链接解释信息

#https://okan.cloud/posts/2021-03-23-visualizing-machine-learning-models/

pdf(file="结果/绝对残差图.pdf", width=6, height=6)

p1 <- plot(mp_rf, mp_svm, mp_glm, mp_gbm, mp_knn, mp_nnet,mp_lasso,)

print(p1)

dev.off()

#绘制四种方法的残差箱线图

pdf(file="结果/箱形图.pdf", width=6, height=6)

p2 <- plot(mp_rf, mp_svm, mp_glm, mp_gbm, mp_knn , mp_nnet, mp_lasso, geom = "boxplot")

print(p2)

dev.off()

p3 <- plot(mp_rf, mp_svm, mp_glm, mp_gbm, mp_knn , mp_nnet, mp_lasso, geom = "histogram")

print(p3)

p4 <- plot(mp_rf, mp_svm, mp_glm, mp_gbm, mp_knn , mp_nnet, mp_lasso, geom = "prc")

print(p4)

#绘制ROC曲线

pred1=predict(mod_rf, newdata=test, type="prob")

pred2=predict(mod_svm, newdata=test, type="prob")

pred3=predict(mod_glm, newdata=test, type="prob")

pred4=predict(mod_gbm, newdata=test, type="prob")

pred5=predict(mod_knn, newdata=test, type="prob")

pred6=predict(mod_nnet, newdata=test, type="prob")

pred7=predict(mod_lasso, newdata=test, type="prob")

roc1=roc(yTest, as.numeric(pred1[,2]))

roc2=roc(yTest, as.numeric(pred2[,2]))

roc3=roc(yTest, as.numeric(pred3[,2]))

roc4=roc(yTest, as.numeric(pred4[,2]))

roc5=roc(yTest, as.numeric(pred5[,2]))

roc6=roc(yTest, as.numeric(pred6[,2]))

roc7=roc(yTest, as.numeric(pred7[,2]))

roc8=roc(yTest, as.numeric(pred8[,2]))

pdf(file="结果/ROC.pdf", width=5, height=5)

plot(roc1, print.auc=F, legacy.axes=T, main="", col="chocolate")

plot(roc2, print.auc=F, legacy.axes=T, main="", col="aquamarine3", add=T)

plot(roc3, print.auc=F, legacy.axes=T, main="", col="bisque3", add=T)

plot(roc4, print.auc=F, legacy.axes=T, main="", col="burlywood", add=T)

plot(roc5, print.auc=F, legacy.axes=T, main="", col="darkgoldenrod3", add=T)

plot(roc6, print.auc=F, legacy.axes=T, main="", col="darkolivegreen3", add=T)

plot(roc7, print.auc=F, legacy.axes=T, main="", col="dodgerblue3", add=T)

plot(roc8, print.auc=F, legacy.axes=T, main="", col="darksalmon", add=T)

legend('bottomright',

c(paste0('RF: ',sprintf("%.03f",roc1$auc)),

paste0('SVM: ',sprintf("%.03f",roc2$auc)),

paste0('GLM: ',sprintf("%.03f",roc3$auc)),

paste0('GBM: ',sprintf("%.03f",roc4$auc)),

paste0('KNN: ',sprintf("%.03f",roc5$auc)),

paste0('NNET: ',sprintf("%.03f",roc6$auc)),

paste0('LASSO: ',sprintf("%.03f",roc7$auc)),

col=c("chocolate","aquamarine3","bisque3",

"burlywood","darkgoldenrod3","darkolivegreen3",

"dodgerblue3","darksalmon"), lwd=2, bty = 'n')

dev.off()

#对四种方法进行基因的重要性分析,得到四种方法基因重要性评分

##此处运行时间较长，每一种运算方式大约半分钟，耐心等待

importance_rf<-variable_importance(

explainer_rf,

loss_function = loss_root_mean_square

)

importance_svm<-variable_importance(

explainer_svm,

loss_function = loss_root_mean_square

)

importance_glm<-variable_importance(

explainer_glm,

loss_function = loss_root_mean_square

)

importance_knn<-variable_importance(

explainer_knn,

loss_function = loss_root_mean_square

)

importance_gbm<-variable_importance(

explainer_gbm,

loss_function = loss_root_mean_square

)

importance_nnet<-variable_importance(

explainer_nnet,

loss_function = loss_root_mean_square

)

importance_lasso<-variable_importance(

explainer_lasso,

loss_function = loss_root_mean_square

)

importance_dt<-variable_importance(

explainer_dt,

loss_function = loss_root_mean_square

)

#绘制基因重要性图形

pdf(file="结果/重要性.pdf", width=7, height=12)

plot(importance_rf[c(1,(ncol(data)-8):(ncol(data)+1)),],

importance_svm[c(1,(ncol(data)-8):(ncol(data)+1)),],

importance_gbm[c(1,(ncol(data)-8):(ncol(data)+1)),],

importance_knn[c(1,(ncol(data)-8):(ncol(data)+1)),],

importance_nnet[c(1,(ncol(data)-8):(ncol(data)+1)),],

importance_lasso[c(1,(ncol(data)-8):(ncol(data)+1)),],

importance_dt[c(1,(ncol(data)-8):(ncol(data)+1)),],

importance_glm[c(1,(ncol(data)-8):(ncol(data)+1)),])

dev.off()

#输出重要性评分最高的基因

geneNum=12 #设置基因的数目

write.table(importance_rf[(ncol(data)-geneNum+2):(ncol(data)+1),], file="结果/核心基因RF.txt", sep="\t", quote=F, row.names=F)

write.table(importance_svm[(ncol(data)-geneNum+2):(ncol(data)+1),], file="结果/核心基因SVM.txt", sep="\t", quote=F, row.names=F)

write.table(importance_glm[(ncol(data)-geneNum+2):(ncol(data)+1),], file="结果/核心基因GLM.txt", sep="\t", quote=F, row.names=F)

write.table(importance_gbm[(ncol(data)-geneNum+2):(ncol(data)+1),], file="结果/核心基因GBM.txt", sep="\t", quote=F, row.names=F)

write.table(importance_knn[(ncol(data)-geneNum+2):(ncol(data)+1),], file="结果/核心基因KNN.txt", sep="\t", quote=F, row.names=F)

write.table(importance_nnet[(ncol(data)-geneNum+2):(ncol(data)+1),], file="结果/核心基因NNET.txt", sep="\t", quote=F, row.names=F)

write.table(importance_lasso[(ncol(data)-geneNum+2):(ncol(data)+1),], file="结果/核心基因LASSO.txt", sep="\t", quote=F, row.names=F)

write.table(importance_dt[(ncol(data)-geneNum+2):(ncol(data)+1),], file="结果/核心基因DT.txt", sep="\t", quote=F, row.names=F)
